# Synergistic Effect of Various Regulatory Factors in Th1/Th2 Balance; Immunotherapeutic Approaches in Asthma

**Published:** 2008-03

**Authors:** Young-Cheol Lee

**Affiliations:** *Department of Herbology, College of Oriental Medicine, Sangji University, Republic of Korea*

**Keywords:** regulatory T cell, Th1/Th2, IL-4, IFN-γ, asthma

## Abstract

The immune balance controlled by T helper 1 (Th1) and T helper 2 (Th2) is crucial for immunoregulation and its imbalance causes various immune diseases including allergic asthma. Therefore, diagnosis of Th1/Th2 balance in autoimmune diseases including asthma is essential for the application of immune balance regulating drugs. Th1/Th2 balance is not only controlled by Th1 cells and Th2 cells, but also by various regulatory factors including regulatory T cells, sexual factor, chemokines, transcription factors, signal transduction pathway (STAT6) etc. From that point of view, conception of “Th1/Th2 balance” resembles the balance of yin and yang which is main concept of Korean traditional medicine for treatment diseases. This article discusses various regulatory factors that influence Th1/Th2. Current research strategies seek to exploit these observations to improve the generation of novel targets for regulating Th1/Th2 balance. The Th1/Th2 balance could be influenced by imunomodulatory drugs (including herbs, prescription and its main components) but this way of therapy needs further evaluation focused on this various factors and synergistic effect.

## INTRODUCTION

### Regulatory T cells diversity & Th1/Th2 balance

‘Naturally occurring’ CD4+ T regulatory cells (nTreg) are derived centrally in the thymus and constitutively express CD25 (the α chain of the IL-2 receptor) and other suppressive molecules including CTLA-4 (1, 2). These cells generally appear to exert suppressive effects by direct cell contact rather than cytokine production. The Foxp3 (forkhead box P3) gene appears to be a critical regulator of the development of this subgroup of CD4+CD25+ Trn cells (3).

At a population level, there has been a parallel rise in both Th1-mediated autoimmune diseases (such as type 1 diabetes, inflammatory bowel disease, multiple sclerosis) and Th2-mediated allergic diseases (4).

At the individual level, there is accumulating evidence that atopy is associated with an increase in both Th1 and Th2 responses (5, 6). Furthermore, Th1 cells also appear to play a role in allergic inflammation in local tissues, failing to counter balance Th2 responses in airways inflammation (7).

These observations lead to the opinion that the autoimmune diseases may develop as a result of a more fundamental failure of underlying immune regulation, rather than a simple skewing of immune response along a Th1/Th2 homeostasis as previously thought.

The regulation of normal and allergic immune responses to allergens in the mucosa is still poorly understood, and the mechanism of specific immunotherapy in normalizing the allergic response to such allergens is currently not clear. Numerous studies have demonstrated alterations in T cell reactivity after allergen immunotherapy, with reduction in Th2 cytokine expression upon allergen stimulation often accompanied by increased expression of the Th1-associated cytokine IFN-γ (8, 9). Recently, attention has focused on IL-10, an immunodulatory cytokine that can down regulate production of both Th1 and Th2 cytokines (10). Several groups have suggested that allergen immunotherapy induces a CD25+ IL-10- producing regulatory T cell subset (11, 12).

Cytokines are of major importance because IL-4 and IL-13 induce the production of IgE by B cells and IL-5 regulates the growth, differentiation, and activation of eosinophils (13). Both IL-4 and IL-5 can directly induce AHR (airway hyperreactivity), airway and blood eosinophilia in asthmatic patients (14-17). The role of Th1-type cytokine IFN-γ in asthma is still a matter of debate: in an earlier study Krug and coworkers described an increased frequency of IFN-γ + T cells in bronchoalveolar lavage fluid from asthmatic compared with control subjects (18), and Hessel and colleagues have early demonstrated that the development of AHR is IFN-γ dependent. However, other investigators have shown an inhibitory effect of IFN-γ on pulmonary allergic responses (19).

nTreg cells constitutively express cytotoxic T lymphocyte-associated antigen 4 (CTLA-4), whereas other types of T cells express it after activation. It has been shown that blockade of CTLA-4 *in vitro* inhibits nTreg cell-mediated suppression (20). Foxp3 is believed to be a master regulator of CD25+ Treg cell development and function. A transcription factor specific for naturally occurring Treg cells is Foxp3 (21), a forkhead box transcription factor that is involved in the induction of the suppressor phenotype of these cells (22).

These cells can also function through induction of inhibitory cytokines, such as transforming growth factor-β (TGF-β) (23). IL-10 was originally described as a cytokine produced by Th2 cells (24). However, it soon became clear that this suppressive cytokine was produced by other cells, including Th1 cells (25).

### Sexual factors & Th1/Th2 balance

Many autoimmune diseases preferentially affect women. The underlying reasons for this gender bias are not clear, but are thought to relate to the effects of sex hormones on the immune system. Males and females appear to show differential responses in many immunological settings. Females were shown to have a more developed thymus, greater resistance to tolerance induction in some animal models, and more pronounced tumor allograft rejection (26, 27). Women have elevated immunoglobulin levels compared to men and an elevated CD4/CD8 T cell ratio in peripheral blood (28). Conversely, women compared to men show a reduced antibodydependent cell-mediated and natural killer (NK) cell cytotoxicity (29, 30).

The heightened immune responsiveness in females may contribute to the greater susceptibility of women to autoimmune diseases such as asthma, multiple sclerosis (MS), rheumatoid arthritis (RA), Grave’s disease, systemic lupus erythematosus (SLE), myasthenia gravis, Sjogren’s syndrome, and Hashimoto’s thyroiditis.

Very few studies of sex differences involving cytokine production *in vivo* have been reported to date. Nevertheless, sex steroids seem to differentially affect Th1 and Th2 cytokine production. Sex steroid hormones are produced by the ovaries and the testes and include estrogens, progestins, and androgens (such as testosterone). Steroid hormones, such as cortisol or dihydoepiandrosterone (DHEA) are produced by the adrenal gland. Protein hormones, such as prolactin, are produced by in the anterior pituitary. Binding sites for sex steroids are present on lymphocytes (31, 32) and they can be metabolized in immunocompetent cells (33), suggesting that sex steroids may affect leukocyte function directly.

Estrogen has effects beyond modulation of sex differentiation, sex function, and its effects on immune cells. Estrogen was shown to directly influence cytokine secretion by CD4 T-cell clones isolated from multiple sclerosis patients (34, 35). Although some studies show contradictory findings, the large majority of literature suggests that androgens and progesterone have immunosuppressive effects, prolactin is stimulatory, and estrogen can be either stimulatory (at low doses) or inhibitory (at higher doses) for immune function (36).

Studies suggest that high levels of 17β-estradiol present during days 14-18 of the menstrual cycle down-regulate the CD3+CD8+cytolytic T cell activity in the uterus (37). In contrast to most findings, a recent human study showing the *in vivo* effects of sex steroids on lymphocyte responsiveness demonstrated that women treated with androgens showed significantly enhanced mitogen-induced IFN-γ/IL-4 ratios and increased TNF-α production (38). Studies of pregnancy have suggested that sex steroids may drive the balance toward Th1 or Th2 cytokine responses. Whitacre *et al*. (39) propose that females are more likely than males to develop a Th1 profile when challenged with an infectious agent. Hamano *et al*. (39) showed that estrogen levels similar to those during pregnancy can stimulate production of the Th2 cytokine, IL-4, from human PBMC (peripheral blood mononuclear cells).

Huber *et al*. (40) showed that testosterone promotes IFN-γ production from CD4 cells and estradiol promotes IL-4 cytokines in spleen cells of C57BL/6 mice. Nevertheless, there is considerable uncertainty about how sex hormones regulate cytokine release and the Th1/Th2 balance. More must be learned about estrogen-regulated genes and the effect of estrogen therapy on promoters for Th1 and Th2 cytokines.

The cross-regulatory nature of the Th1/Th2 balance suggests that maturing lymphocytes can change their secretory pattern over time and specific sex steroids may produce different modulatory effects depending on the cytokine profile of the cells at that moment in time (41).

Lymphocytes from female mice were found to produce higher levels of IFN-γ after immune stimulation than lymphocytes from males, both *in vivo* and *in vitro* (42, 43). The finding that CD8+ T cells express estrogen receptors is consistent with the estrogen-enhanced expression of the IFN-γ gene, since it was reported that CD8 cells were responsible for the majority of IFN-γ production in antigen- or mitogen-stimulated cultures of murine lymph node cells and splenocytes.

Other steroid hormones, such as cortisol or dihydoepiandrosterone (DHEA) are produced by the adrenal gland. And these hormones may affect leukocyte function directly (45). Sex steroids may produce different modulatory effects depending on the cytokine profile of the cells. However, it is not clear how sex steroids regulate cytokine release and the Th1/Th2 balance.

### Chemokines & Th1/Th2 balance

Chemokines are chemotactic cytokines produced by a wide variety of cells to attract the relevant lymphocytes to the appropriate sites in the body. Th1 and Th2 cells express different chemokine receptor profiles. Th1 cells are thought to preferentially express the CC chemokine receptors CCR1, CXCR3, and CCR5 (46, 47).

Chemokines, including thymus and activation-regulated chemokine (TARC), are important for the regulation of inflammation and IgE synthesis. Because TARC is responsible for trafficking of Th2 lymphocytes into sites of allergic inflammation, increases in TARC promote Th2 trafficking and, through this activity, increase IgE sensitization and asthma severity (48).

In a recent study (49), the regulation of chemokine production from Th1 or Th2 lymphocyte populations indicated that there was a differential chemokine response that depended on regulation by the STAT6 (signal transducers and activators of transcription 6)-mediated pathways.

The role of chemokines in allergic disease progression is evident at multiple levels, including differential leukocyte recruitment and local cellular activation.

Therefore, chemokines could control and direct the migration and activation of various leukocyte populations. Targeting chemokines should lead to new ways of controlling the inflammatory immune response.

### Transcription factors & Th1/Th2 balance

Several factors determine the fate of activated T cells, type of antigen presenting cells, co-stimulatory molecules, and most importantly, cytokines present in the local environment of the cell at the time of stimulation. T-box expressed in T cells (T-bet) and GATA-binding protein 3 (GATA-3) are two major T helper-specific transcription factors that regulate the expression of Th1 or Th2 cytokine genes and play a crucial role in T-helper cell differentiation. T-bet, a newly discovered Th1-specific transcription factor is thought to initiate Th1 development while inhibiting Th2 cell differentiation (50, 51). GATA-3 is a member of the GATA family of zinc finger proteins (so-called because they bind to consensus DNA sequence, A/T; GATA A/G), and plays a pivotal role in the development of the Th2 phenotype while inhibiting Th1 cells (52, 53).

Among the many signals that influence the development of Th cells, two have been suggested to be master regulators of Th1 and Th2 differentiation, respectively, expression of the transcription factors, T-bet and GATA-3 (54).

Takumi *et al*. (55) demonstrated that overexpression of T-bet and GATA-3 regulates the development of allergeninduced airway remodeling. Serum immunoglobulin levels and cytokine analysis showed that the Th1/Th2 balance shifted toward Th1 in T-bet-*tg* mice and toward Th2 in GATA-3-*tg* mice after chronic allergen exposure. It shows that development of airway remodeling is regulated by the lung Th1/Th2 bias induced by GATA-3 and T-bet.

### Transduction pathway & Th1/Th2 balance

Signal transducer and activator of transcription molecules, or STATs, have been identified as important regulators in the transduction pathways of the interferon molecules (56). Especially, Th2 cytokines, IL-4 and IL-13, activate STAT1 signaling pathways across multiple cell lines (25). STAT1 in particular is responsive to interferon-γ, leading to the transcription of multiple genes, including principally ICAM-1 and IRF-1, both of which have been implicated in asthma.

STAT6 is another pathway in the family of STAT that has also been implicated in the development of allergic disease and asthma. Its primary activators are IL-4 and IL-13. Many have hypothesized that STAT6 might have a role in asthma, based upon its actions and location on chromosome 12 (57).

STAT6 is activated by the Th2-type cytokines, IL-4 and IL-13, it is clear that the chemokines can be controlled almost exclusively by the level of expression of the Th2-type cytokines (58).

Allergic airways disease is initiated and perpetuated by Th2 cytokines IL-4 and IL-13, each of which induces activation of the STAT-6 transcription factor. McCusker *et al*. (59) showed that the clinical manifestations of acute asthma are STAT-6 dependent, and STAT-6 is a target for drug development in allergic airways disease.

## CONCLUSIONS

**Figure 1:**
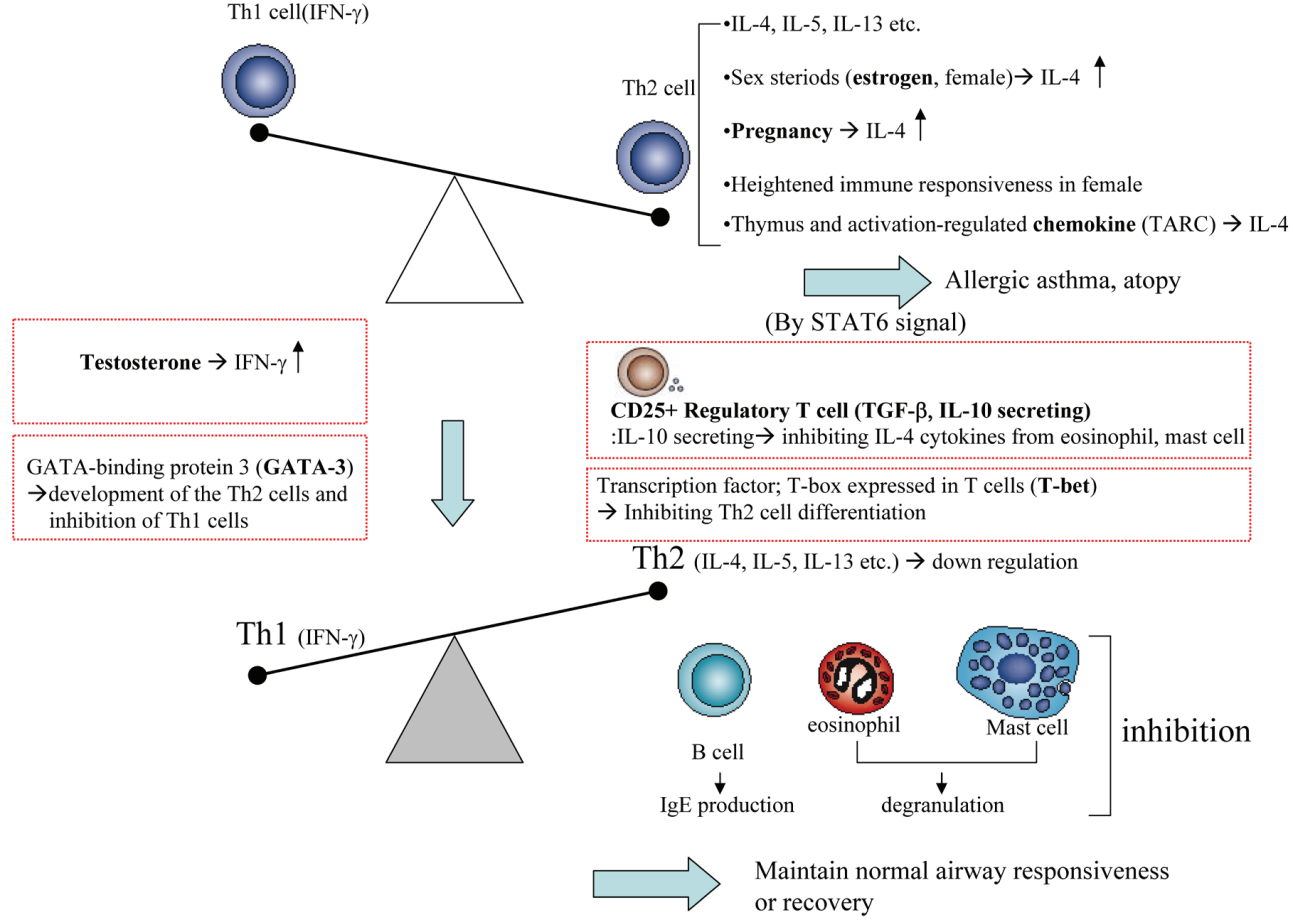
Various regulatory factors (CD25+, sexual steroids, chemokines etc.) in Th1/Th2 balance.

Both naturally occurring and inducible CD4+CD25+ regulatory T cells inhibit these inappropriate immune responses in Th1/Th2 balance. Sexual hormones especially testosterone promotes IFN-γ production from CD4 cells isolated from multiple sclerosis patients and estradiol promotes IL-4 cytokines in spleen cells of C57BL/6 mice. Chemokines, including thymus and activation-regulated chemokine (TARC), are responsible for trafficking of Th2 lymphocytes into sites of allergic inflammation, that depended on regulation by the STAT6 (signal transducers and activators of transcription 6)-mediated pathways and increases in TARC promote Th2 trafficking. T-box expressed in T cells (T-bet) is thought to initiate Th1 development while inhibiting Th2 cell differentiation. GATA-binding protein 3 (GATA-3) plays a pivotal role in the development of the Th2 phenotype while inhibiting Th1 cells (Figure 1).

Th1/Th2 cytokine imbalance contributes to the etiology and pathogenesis of asthma, understanding these mechanisms will provide novel targets for therapeutic drugs.

The Th1/Th2 balance could be influenced by imunomodulatory drugs (including herb medicines, prescrioption and its main components) but this way of therapy needs further evaluation.

**Figure 2:**
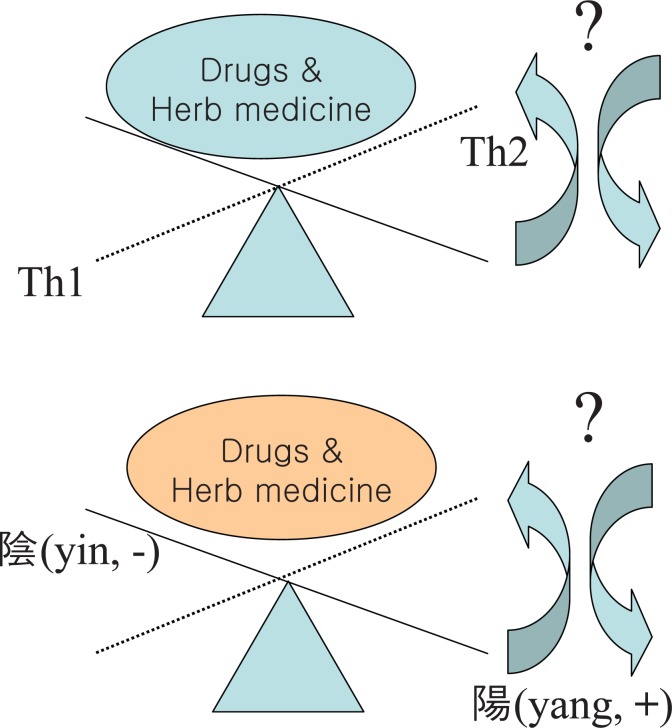
(yin and yang) homeostasis & Th1 Th2 balance for seek novel targets by regulating Th1/Th2 balance.

The future will probably bring us novel targets for regulating Th1/Th2 balance. Modification of lymphocytes, infusions of blocking antibodies, production of lymphocytes targeted towards bound with Th2 or Th1 cytokines could be potentially effective (Figure 2).
